# Lusutrombopag for thrombocytopenia in Chinese patients with chronic liver disease undergoing invasive procedures

**DOI:** 10.1007/s12072-022-10421-9

**Published:** 2022-10-18

**Authors:** Zhenbin Ding, Hong Wu, Yongyi Zeng, Ming Kuang, Wei Yang, Zhiqiang Meng, Yajin Chen, Chunyi Hao, Shubing Zou, Huichuan Sun, Chang Liu, Kecan Lin, Guoming Shi, Xiaoying Wang, Xiutao Fu, Rongxin Chen, Yi Chen, Ruifang Liang, Takeshi Kano, Huiyan Pan, Suna Yang, Jia Fan, Jian Zhou

**Affiliations:** 1 Department of Liver Surgery and Transplantation, Liver Cancer Institute, Zhongshan Hospital, Fudan University, Shanghai, 200032 China; 2 Department of Liver Surgery and Liver Transplantation, West China Hospital of Sichuan University, Chengdu, 610041 Sichuan China; 3 The United Innovation of Mengchao Hepatobiliary Technology Key Laboratory of Fujian Province, Mengchao Hepatobiliary Hospital of Fujian Medical University, Fuzhou, 350025 Fujian China; 4 Department of Liver Surgery, The First Affiliated Hospital of Sun Yat Sen University, Guangzhou, 510080 Guangdong China; 5 Department of Hepatobiliary Surgery, The First Affiliated Hospital of Xi’an Jiaotong University, Xi’an, 710061 Shanxi China; 6 Minimally Invasive Therapy Center, Fudan University Shanghai Cancer Center, Shanghai, 200032 China; 7 Department of Hepatobiliary Surgery, Sun Yat Sen Memorial Hospital of Sun Yat Sen University, Guangzhou, 510120 Guangdong China; 8 Department of Hepatobiliary Surgery, Beijing Cancer Hospital, Beijing, 100142 China; 9 Department of Hepatobiliary Surgery, The Second Affiliated Hospital of Nanchang University, Nanchang, 330006 Jiangxi China; 10 Liver Cancer Institute, Zhongshan Hospital of Fudan University, Shanghai, 200032 China; 11 Eddingpharm Co, Ltd, Unit 122-129, Building A3, No. 700, Wanrong Road, Shanghai, China; 12 Shionogi & Co, Ltd, 3-13, Imabashi 3-chome, Chuou-ku, Osaka, 541-0042 Japan; 13 Key Laboratory of Carcinogenesis and Cancer Invasion (Fudan University), Ministry of Education, Shanghai, 200032 China

**Keywords:** Thrombopoietin receptor agonist, Clinical study, Platelet, Thrombosis, Hemorrhage, Placebo, Cirrhosis, Hepatitis B, Dose-stopping, Platelet transfusion

## Abstract

**Purpose:**

Probing efficacy and safety of lusutrombopag in Chinese chronic liver disease (CLD) and severe thrombocytopenia (PLT < 50 × 10^9^/L) patients undergoing elective invasive procedures.

**Methods:**

In this double-blind, parallel-group phase 3 study, 66 patients with CLD and severe thrombocytopenia were randomized 2:1 to lusutrombopag or placebo arm treatment regimens for seven days at 9 centers in China. Responders (PLT ≥ 50 × 10^9^/L that increased to ≥ 20 × 10^9^/L from the baseline and not received rescue therapy for bleeding) on Day 8 (the day after seven-day treatment) were assessed. PLT ≥ 50 × 10^9^/L on or after Day 8 and within 2 days before invasive procedure (alternative criteria for not requiring platelet transfusion) were also analyzed. Adverse events (AEs) were recorded.

**Results:**

The proportion of responders on Day 8 was evidently higher (*p* = 0.0011) in the lusutrombopag group (43.2%, 19/44) versus placebo (4.5%, 1/22). And 72.7% (32/44) patients receiving lusutrombopag met the alternative criteria for not requiring platelet transfusion, while 18.2% (4/22) in the placebo group. The median maximum PLT in lusutrombopag group increased to 80.5 × 10^9^/L, and median time to reach maximum was 14.5 days. Compared with placebo, the lusutrombopag group had a lower incidence of bleeding events (6.8% versus 13.6%), and only one patient had thrombotic-related AE. Overall, the incidence of treatment-emergent AEs was comparable between two groups.

**Conclusions:**

Lusutrombopag was effective in raising PLT, diminishing platelet transfusion requirement, and documented a safety profile like the placebo in CLD and severe thrombocytopenia patients in a Chinese cohort undergoing elective invasive procedures. Chinese clinical trial registration number: CTR20192384.

**Supplementary Information:**

The online version contains supplementary material available at 10.1007/s12072-022-10421-9.

## Introduction

Chronic liver disease (CLD) is recognized as a major public health problem worldwide. It is estimated that 1.5 billion people suffer from CLD globally, and its prevalence is increasing [1]. Close to 300 million individuals are impacted by liver disease in China, especially hepatitis B and cirrhosis, which has brought a huge burden to society [2]. Thrombocytopenia is a ubiquitous condition in patients with CLD. Around 76% of CLD patients were believed to have some degree of thrombocytopenia, with a higher incidence observed in cirrhosis patients [3]. The severity of thrombocytopenia correlates with both severity as well as to long-term outcomes of liver disease [4]; more importantly, severe thrombocytopenia (< 50 × 10^9^/L) is a predictive indicator of major bleeding or re-bleeding in perioperative settings, which brings great challenges to surgical management [5].

Platelet transfusion is the fastest and gold-standard treatment for thrombocytopenia in patients receiving invasive procedures. Refer to the blood transfusion guidelines to recommend using platelet counts (PLT) < 50 × 10^9^ /L as the standard to determine whether preoperative platelet transfusion is required [6, 7]. However, clinicians tried to avoid its frequent use due to the disadvantages such as safety risks of transfusion, donor shortage and high costs, especially in China. Splenectomy and splenic artery embolization are also effective in patients with CLD and thrombocytopenia. However, concerns remain regarding the serious complications after splenectomy and the recurrence of thrombocytopenia after splenic embolization [3, 4].

Recently, more clinical evidence has suggested that small-molecule oral thrombopoietin receptor agonists (TPO-RAs) can raise PLT. TPO-RAs was suggested as an effective option to transfusions of platelets in the treatment of CLD with thrombocytopenia in the United States [8]. Currently, only avatrombopag was approved for CLD and thrombocytopenia patients undergoing elective invasive procedures in China [9]. However, avatrombopag has a certain risk of drug-drug interactions, and its blood concentration is easily affected by many factors such as diet. Therefore, there is an urgent need for more choices to meet the needs of these patients [10].

Lusutrombopag, a novel second-generation oral TPO-RA, can also act on receptors of TPO expressed in megakaryocytes to activate the differentiation and proliferation of megakaryocytes and promote thrombocytopoiesis [11]. Studies L-PLUS 1 and PLUS 2 were two randomized, double-blind, phase 3 trials; they demonstrated that lusutrombopag could effectively raise PLT and lower the requirements for platelet transfusion [12, 13]. Moreover, there is an absence of restrictions on food and clinically significant interactions between drugs [14, 15]. Unfortunately, there is no evidence-based data for lusutrombopag in a Chinese cohort yet. None of the Chinese patients had the opportunity to participate in the two studies and try the drug treatment of lusutrombopag. As we all know, as a high incidence area of hepatitis B virus infection, China is the country with the highest incidence rate and mortality of liver cancer in the world, 80% of which is related to hepatitis B. This study was the first phase 3 clinical trial of lusutrombopag (S-888711) in China, aiming to evaluate its efficacy and safety in CLD and thrombocytopenia patients undergoing elective invasive procedures.

## Methods

### The design of the study and treatment

This randomized, multicenter, placebo-controlled, double-blind, phase 3 study was conducted in 9 sites in China.

The study entailed three periods: screening (up to 4 weeks), treatment (1 week), and post-treatment (4 weeks) (Supplementary material 1). Screening of likely patients who submitted written informed consent was done for PLT to scrutinize their eligibility, and the results were regarded as baseline data. Random allocation of eligible patients was done to lusutrombopag or placebo arm in a 2:1 ratio for the intake of one tablet a day of the study drug (3 mg of lusutrombopag or placebo)from first day of the treatment period for seven days. PLT was evaluated on Day 8 (the day after 7-day treatment) for primary endpoint analysis and then tested on Days 10, 15, 17, 21, 28, and 35. The planned procedure that was invasive was performed between days 9 and 15. If the PLT was < 50 × 10^9^/L, preprocedural platelet transfusion was allowed.

### Participants

The eligibility criteria were: patients who were 18 years or more when signing the informed consent with Child–Pugh class A or B liver disease, or with Child–Pugh class C liver disease but can be hospitalized at least between days 5 and 10 and were undergoing for elective invasive treatment probably needing platelet transfusion, and the PLT was < 50 × 10^9^/L at baseline. The exclusion of a few procedures was done inclusive of thoracotomy, laparotomy, open-heart surgery, craniotomy, organ or partial organ resection. All patients documented an Eastern Cooperative Oncology Group performance in grades of 0 or 1 and were infertile or consented to the use of appropriate contraception.

Patients who had any other causes of thrombocytopenia, any solid malignant tumor required systemic chemotherapy or with metastasis, past or present thrombotic or hemorrhagic diseases, or with a history of liver transplantation were excluded (Supplementary material 2).

### Assessments

The percentage of responders (patients with PLT ≥ 50 × 10^9^/L documenting an increase of ≥ 20 × 10^9^/L from baseline and not received rescue therapy for bleeding) on Day 8 constituted the primary efficacy endpoint. As the alternative criteria for not requiring platelet transfusion in this study, the key secondary efficacy endpoint was the percentage of patients with PLT ≥ 50 × 10^9^/L on or after Day 8 and under two days prior to the day of the procedure. Other secondary efficacy endpoints were the percentage of patients who (a) met the criteria for a responder at any time during the study; (b) needed rescue treatment for bleeding at any time in the study period; (b) the time course change in PLT; (d) units (dose) of transfused platelets and frequency of the platelet transfusion in the study timeframe.

For safety assessments, AEs (adverse events) and AEs of special interest (bleeding- and thrombosis-related AEs) were evaluated. The WHO Bleeding Scale at these time points: in the period of screening; during randomization; day 8; 3–10 days post-procedure; and day 35 (premature termination or at stopping the study drug) was employed to assess the bleeding severity. Furthermore, the protocol also was inclusive of imaging assessments such as ultrasonography, Doppler ultrasonography, computed tomography, or magnetic resonance imaging to score thrombotic events during screening, 6 ± 3 days following the invasive procedure, and premature termination or at stopping the drug under study.

### Statistical analysis

Based on the previous phase 3 studies (L-PLUS 1 study, L-PLUS 2 study), the percentage of patients in the lusutrombopag and placebo groups meeting the primary endpoint was estimated at 41.9% and 3.4%, respectively. A sample size of 54 patients: the lusutrombopag group (*n = *36) and the placebo group (*n = *18) was required to have at least 90% power for scoring a superiority difference of 0 between both groups at 0.05 two-sided significance level. Taking into account the estimated drop-out rate of approximately 20%, the total sample size was 66: the lusutrombopag group (*n = *44) and placebo(*n = *22). For the primary endpoint, the proportion of responders was computed employing Fisher’s exact test, and inter-group comparison was made with the Cochran–Mantel–Haenszel test with PLT at screening as factors for stratification.

For efficacy, the population under primary analysis was the FAS (full analysis set) or all patients who were randomized based on the intention-to-treat principle, while the safety analysis entailed the use of the safety set (SS) that was all randomized patients receiving a minimum of one study drug.

SAS (V 9.4, SAS Institute, USA) was employed for all analyses with significance at a *p* value < 0.05. The MedDRA (Version 23.0) terms were followed for coding the AEs, followed by tabulation for each treatment group by system organ classes and Preferred Term.

## Results

This work was inclusive of 66 patients randomized into the lusutrombopag group (*n = *44) and placebo (*n = *22), which took place from July 2020 to June 2021. A total of 64 patients (44 lusutrombopag, 20 placebo) completed study drug administration (3 mg, for seven days consecutive, and once daily). Two patients in the placebo group missed 1 or 3 days of dosing due to study withdrawal or loss of the medication. Additionally, 59 patients completed the study (40 lusutrombopag, 19 placebo) (see Supplementary material 3 for a complete trial profile).

Table 1 documents the demographic and baseline clinical traits of the FAS. Overall, the baseline characteristics in both groups were well balanced. The mean age was 55.9 ± 10.08 years, 66.7% of the patients were male, 80.3% of the patients were categorized with Child–Pugh A liver disease and 19.7% with Child–Pugh B liver disease. Most patients suffered from chronic hepatitis B, and the mean duration of CLD was 113.77 ± 116.729 months. The most common type of invasive procedure was transcatheter arterial embolization/transhepatic arterial infusion/transcatheter arterial chemoembolization (TAE/TAI/TACE).

**Table 1:** Baseline demographic and clinical characteristics

	Lusutrombopag	Placebo	Total
	*n = *44	*n = *22	*n = *66
Sex			
Male	31 (70.5)	13 (59.1)	44 (66.7)
Female	13 (29.5)	9 (40.9)	22 (33.3)
Age (years)	56.3 (10.75)	55.2 (8.78)	55.9 (10.08)
Type of liver disease			
Hepatitis B	37 (84.1)	20 (90.9)	57 (86.4)
Hepatitis C	1 (2.3)	0 (0.0)	1 (1.5)
Alcoholic hepatitis	1 (2.3)	0 (0.0)	1 (1.5)
Other	5 (11.4)	2 (9.1)	7 (10.6)
Mean duration of CLD (months)	109.73 (117.719)	122.05 (117.249)	113.77 (116.729)
Child–Pugh class			
A	37 (84.1)	16 (72.7)	53 (80.3)
B	7 (15.9)	6 (27.3)	13 (19.7)
Baseline PLT (× 10^9^/L)	38.0 (7.74)	37.1 (7.58)	37.7 (7.64)
< 35	13 (29.5)	7 (31.8)	20 (30.3)
≥ 35	31 (70.5)	15 (68.2)	46 (69.7)
Invasive procedure^a^	39 (88.6)	19 (86.4)	58 (87.9)
RFA	7 (15.9)	2 (9.1)	9 (13.6)
TAE/TAI/TACE	17 (38.6)	11 (50.0)	28 (42.4)
EUS FNA	0 (0.0)	2 (9.1)	2 (3.0)
LRA	1 (2.3)	0 (0.0)	1 (1.5)
Endoscopic polypectomy	1 (2.3)	0 (0.0)	1 (1.5)
EVL	3 (6.8)	2 (9.1)	5 (7.6)
Percutaneous needle biopsy	0 (0.0)	1 (4.5)	1(1.5)
Other	10 (22.7)	1 (4.5)	11 (16.7)

Data are presented as *n* (%) or mean ± standard deviation

*PLT* platelet counts, *RFA* radiofrequency ablation, *TAE* transcatheter arterial embolization, *TAI* transhepatic arterial infusion, *TACE* transcatheter arterial chemoembolization, *EUS FNA* endoscopic ultrasonography-guided fine-needle aspiration, *LRA* laparoscopic radiofrequency ablation, *EVL* endoscopic variceal ligation

^a^Calculated using the actual invasive procedure

### Primary efficacy endpoint

**Fig. 1:**
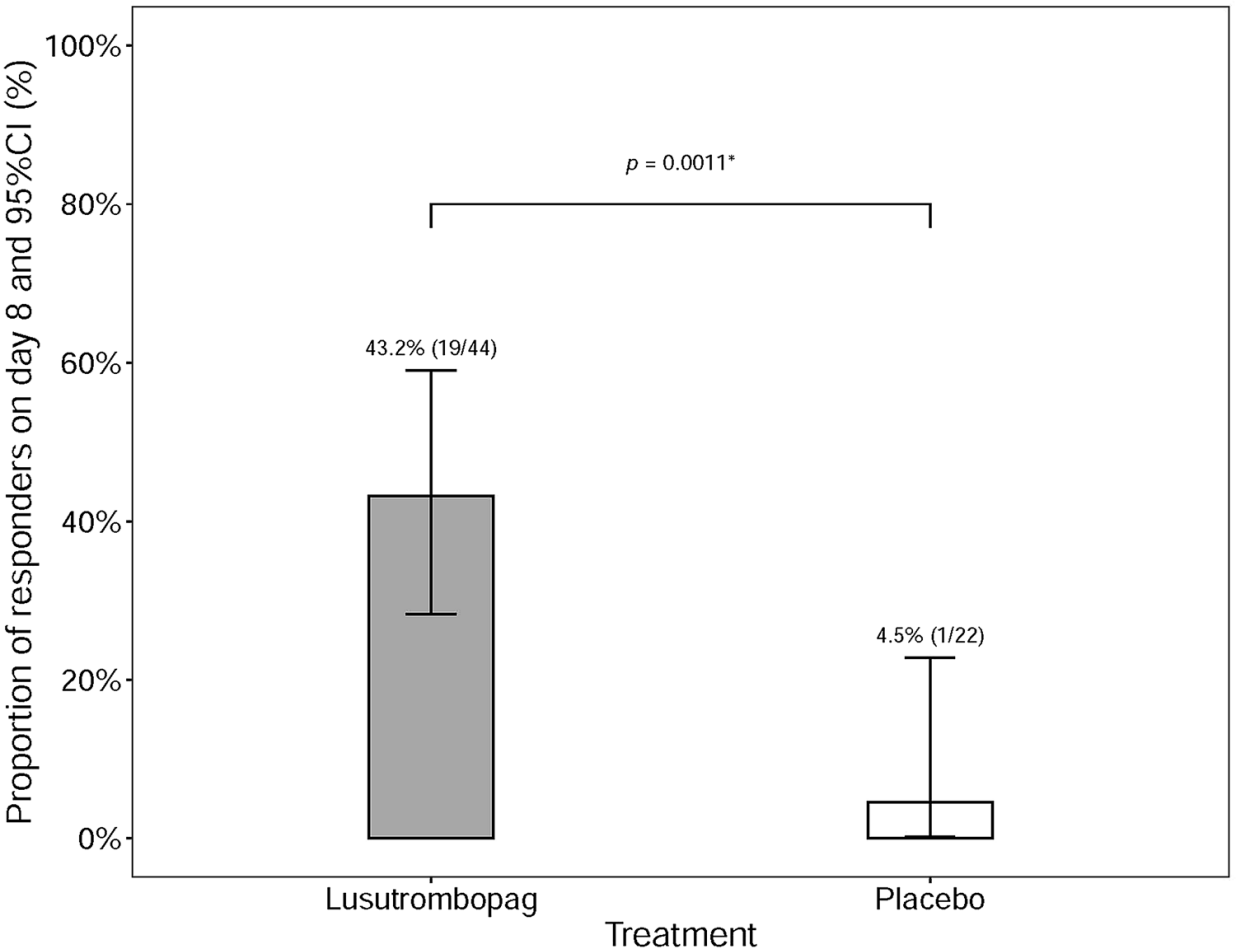
Percentage of responders in the placebo and lusutrombopag groups on Day 8 in FAS population. Responder is the patient with PLT ≥ 50 × 10^9^/L that increased to ≥ 20 × 10^9^/L from the baseline with no rescue therapy for bleeding. *Cochran–Mantel–Haenszel test

The proportion of responders on Day 8 was 43.2% (19/44) in the group receiving lusutrombopag and 4.5% (1/22) in the control placebo group. Between these groups, the proportion difference was statistically significant at 38.6% (*p* = 0.0011) (Fig. 1).

**Fig. 2:**
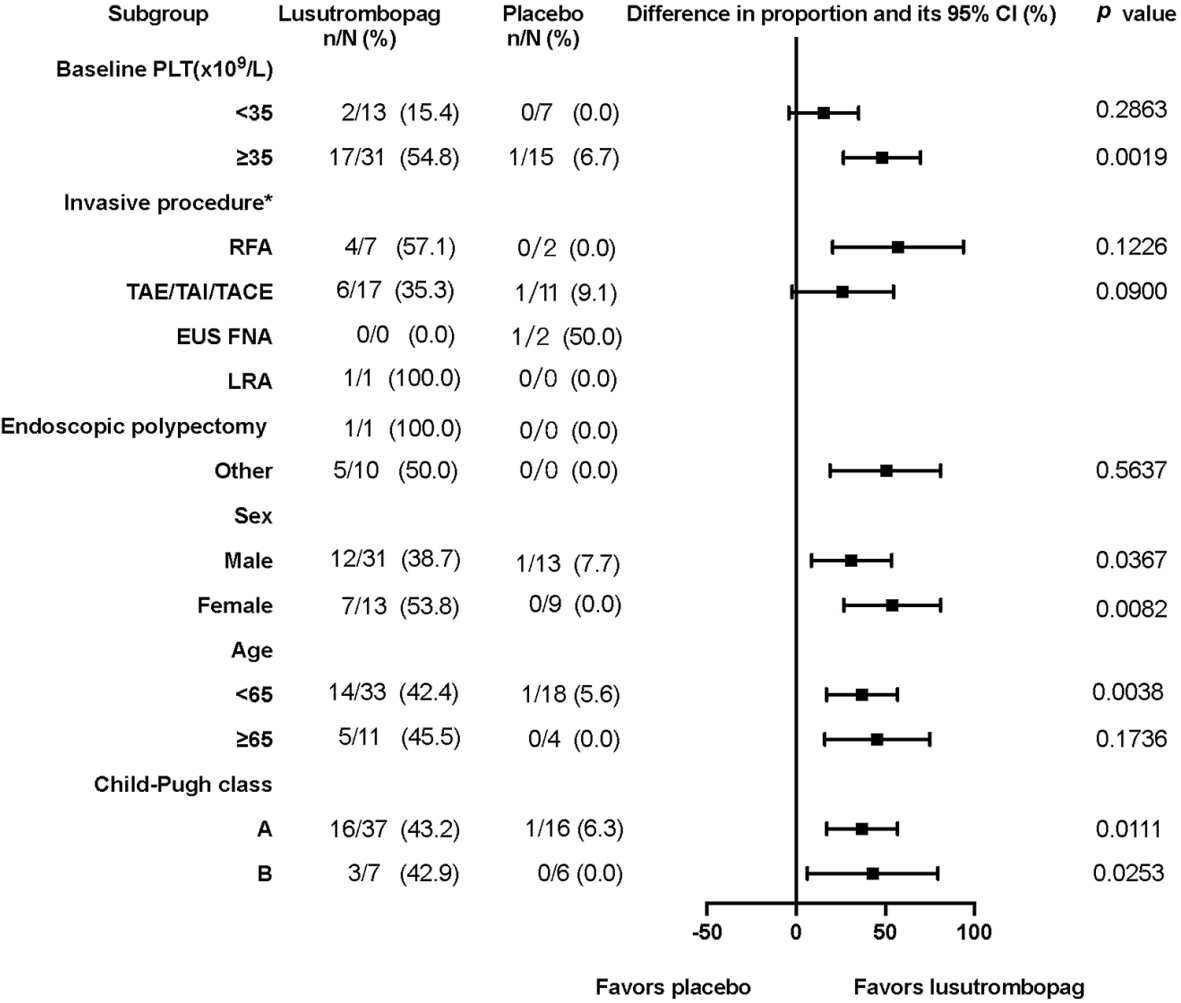
Subgroup analysis of the percentage of responders in the placebo and lusutrombopag groups on Day 8 in FAS population. Data are presented as number of patients/ total number of subgroups and percentage of patients. Responder is the patient with PLT ≥ 50 × 10^9^/L that increased to ≥ 20 × 10^9^/L from the baseline with no rescue therapy for bleeding. * The actual invasive procedure. *PLT* platelet counts, *RFA* radiofrequency ablation, *TAE* transcatheter arterial embolization, *TAI* transhepatic arterial infusion, *TACE* transcatheter arterial chemoembolization, *EUS FNA* endoscopic ultrasonography-guided fine-needle aspiration, *LRA* laparoscopic radiofrequency ablation

The median values of PLT on Day 8 (the day after 7-day treatment) were 61.5 × 10^9^/L and 41.0 × 10^9^/L; and the proportion of patients with PLT ≥ 50 × 10^9^/L on Day 8 was 68.2% and 13.6%; and the proportion of patients with PLT increased ≥ 20 × 10^9^/L from baseline on Day 8 were 43.2% and 4.5% in the lusutrombopag and placebo groups, respectively. Additionally, the subgroup analysis showed a tendency for lusutrombopag superior to placebo in the proportion of responders on Day 8 (Fig. 2).

### Secondary efficacy endpoints

The proportion of patients with PLT ≥ 50 × 10^9^/L on or after Day 8 and within 2 days before procedure was 72.7% (32/44) in the lusutrombopag group and 18.2% (4/22) in the placebo group. The difference of the proportion between the two groups was statistically significant (*p < *0.0001). In addition, significantly more patients who were randomly assigned to lusutrombopag than placebo met the criteria for responder at any time over study period. These results are listed in Table 2.

**Table 2:** Secondary efficacy endpoints in the lusutrombopag and placebo groups

	Lusutrombopag	Placebo
	*n = *44	*n = *22
The key secondary efficacy endpoint		
PLT ≥ 50 × 10^9^/L on or after Day 8 and within two days before the procedure	32 (72.7)	4 (18.2)
*p* value in CMH test	< 0.0001	
Other secondary efficacy endpoints		
Met the criteria for responder at any time	36 (81.8)	12 (54.5)
Required rescue therapy for bleeding at any time	0 (0)	0 (0)
Received platelet transfusion	0 (0)	2 (9.1)^a^
The change from baseline in platelet count over time		
Median maximum PLT (× 10^9^/L)	80.5	60.0
Median maximum increase of PLT from baseline (× 10^9^/L)	42.0	24.0
Median time to reach maximum PLT (days)	14.5	27.0

Data are presented as *n* (%) or mean ± standard deviation

^a^Two patients in the placebo group received one platelet infusion before the procedure. The infusion dose was one dose and the reason for the infusion was that the pre-procedure PLT was < 50 × 10^9^/L

**Fig. 3:**
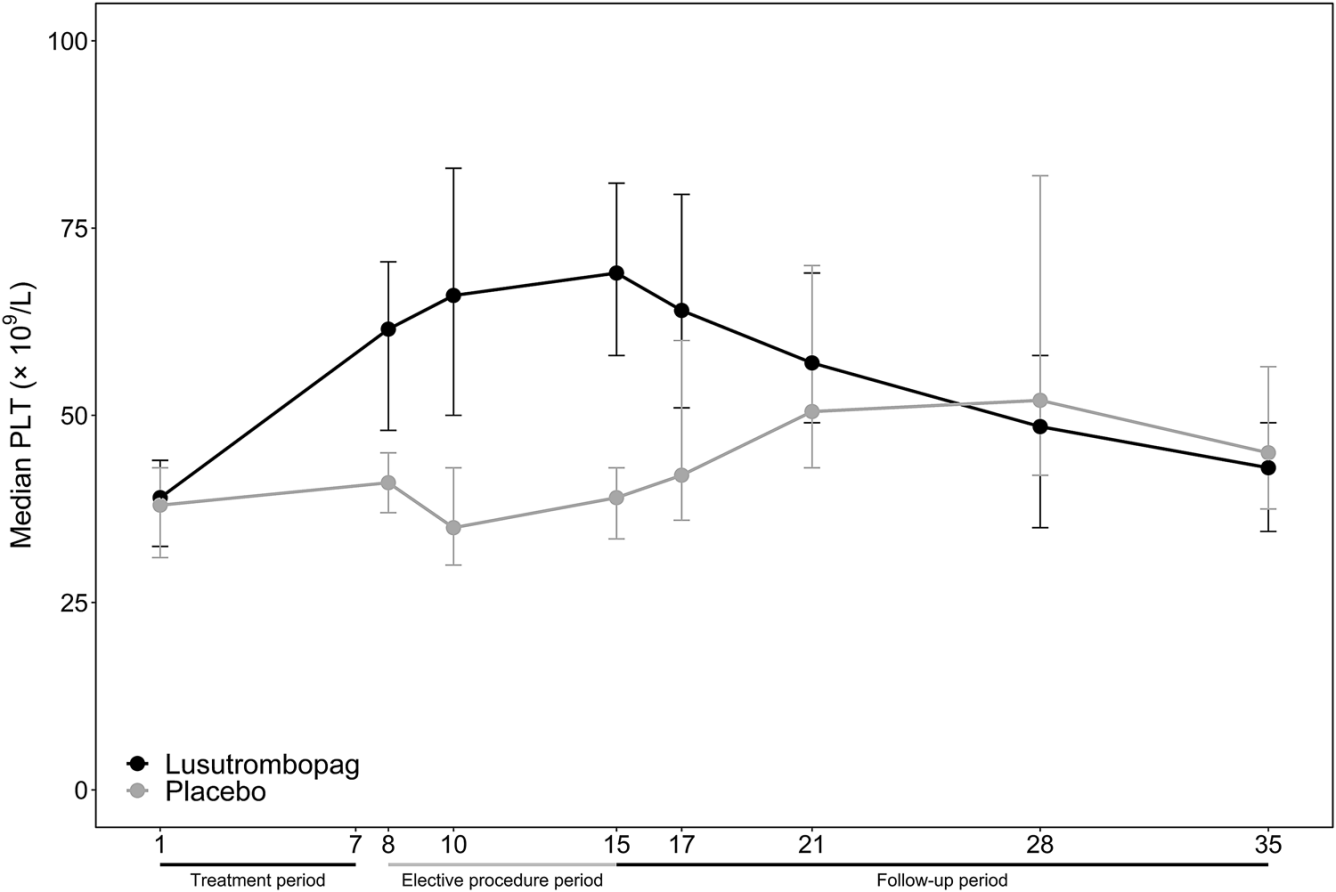
Median PLT in the lusutrombopag and placebo groups over time (error bars indicate standard deviation). Error bars indicate 25th percentile and 75th percentile. *PLT* platelet counts

The median maximum PLT were 80.5 × 10^9^/L and 60.0 × 10^9^/L; the median maximum increase of PLT from baseline were 42.0 × 10^9^/L and 24.0 × 10^9^/L, and the median time to reach the maximum PLT were 14.5 and 27.0 days in the lusutrombopag and placebo groups, respectively (Fig. 3). Moreover, the increase in PLT occurred before invasive procedures in the lusutrombopag group, while the increase of PLT occurred following invasive procedures in the placebo group. The median PLT returned to baseline within 35 days.

### Safety

No patients died or discontinued the study drug because of AEs in the lusutrombopag group. Just one patient in the placebo group experienced AEs leading to study drug discontinuation (fever; a decrease in leukocytes, lymphocytes, and neutrophils). The overall incidence of TEAEs was 84.1% (37/44) in the lusutrombopag group and 90.9% (20/22) in the placebo group (Table 3). Most of the TEAEs were mild or moderate in severity. The incidence of TEAEs after invasive procedures was higher than that before procedures.

**Table 3:** Incidence of AEs in the lusutrombopag and placebo groups

	Lusutrombopag	Placebo
	*N = *44	*N = *22
TEAEs	37 (184), 84.1%	20 (127), 90.9%
TEAEs with an incidence of ≥ 5%^a^	35 (133), 79.5%	19 (85), 86.4%
White blood cell count decreased	13 (17), 29.5%	9 (13), 40.9%
Abdominal pain	10 (13), 22.7%	4 (4), 18.2%
Poor appetite	8 (9), 18.2%	3 (3), 13.6%
AST increased	7 (7), 15.9%	4 (4), 18.2%
Blood bilirubin increased	7 (7), 15.9%	5 (6), 22.7%
Postoperative fever	7 (7), 15.9%	3 (3), 13.6%
Blood unconjugated bilirubin increased	6 (6), 13.6%	2 (2), 9.1%
Pre-procedure TEAEs	18 (36), 40.9%	11 (32), 50.0%
Postoperative TEAEs	33 (148), 75.0%	17 (95), 77.3%
Drug-related TEAEs	5 (7), 11.4%	4 (12), 18.2%
Significant AEs^b^	22 (49), 50.0%	12 (29), 54.5%
Treatment-related significant AEs	2 (2), 4.5%	2 (2), 9.1%
SAEs	3 (4), 6.8%	0 (0), 0.0%
Fatal SAEs	0 (0), 0.0%	0 (0), 0.0%
Non-fatal SAEs	3 (4), 6.8%	0 (0), 0.0%
Drug-related SAEs	0 (0), 0.0%	0 (0), 0.0%
Thrombosis-related AEs	1 (1), 2.3%	0 (0), 0.0%
Bleeding-related AEs	3 (3), 6.8%	3 (4), 13.6%
Treatment termination-related AEs	0 (0), 0.0%	1 (4), 4.5%
Study termination-related AEs	1 (1), 2.3%	0 (0), 0.0%

The presentation of data is in the form of the patient number (number of events) and percentage

*TEAE* treatment-emergent adverse event; *AST* aspartate aminotransferase; *AE* adverse events; *SAE* serious adverse event

^a^Only list the top 5 most common TEAEs in the lusutrombopag group

^b^Significant AEs: AEs and obviously abnormal hematological or other laboratory tests that lead to directed medical treatment (such as drug withdrawal, dose reduction, and symptomatic treatment), except for SAE

A total of 11.4% of patients in the lusutrombopag group and 18.2% of patients in the placebo group experienced drug-related AEs. All were mild or moderate in severity and the incidence of each drug-related AE was less than 5%. Three patients in the lusutrombopag group experienced SAEs (hepatic encephalopathy and coagulopathy; pyrexia; acute cholecystitis), all of which were non-fatal and not related to the study drug.

Bleeding- and thrombosis-related AEs were reported in very few patients over the study period (Table 3). There were three bleeding-related AEs in three patients in the lusutrombopag group (fecal occult blood occurred after the procedure; intraoperative bleeding; bleeding nose occurred before the procedure and after the first administration) and four events in three patients in the placebo group (positive urine occult blood occurred after the procedure; urinary occult blood positive occurred before the procedure and after the first administration; bleeding from the puncture point occurred after the procedure; skin ecchymosis occurred after the procedure). All the bleeding-related AEs were mild or moderate in severity, and no patients received rescue therapy for bleeding throughout the study. Only one patient in the lusutrombopag group experienced thrombosis-related AEs, which was brachiocephalic vein thrombosis, and was considered mild and not related to the study drug.

## Discussion

This phase 3 study showed that the effect of lusutrombopag (3 mg, for seven days consecutive, and once daily) was more significant than that of placebo in raising PLT and diminishing platelet transfusion requirement in adult patients of China with severe thrombocytopenia and CLD being treated by invasive elective procedures. Further, its safety profile was on the similar lines as the placebo, with no increase in the risk of thrombosis-related AEs. The overall results of the study conducted in China are consistent with the previous studies L-PLUS 1 and 2 [12, 13]. Therefore, more patients with CLD and thrombocytopenia will benefit from lusutrombopag, including not only HCV patients with thrombocytopenia undergoing EVL or endoscopic biopsy, but also HBV patients with thrombocytopenia undergoing invasive treatment such as TACE or RFA.

L-PLUS 1 was a randomized, double-blind, placebo-controlled phase 3 clinical trial, which showed that the proportion of patients in lusutrombopag group agrees with the primary endpoint (no pre-procedure requirement of platelet transfusions) is significantly higher than that in placebo group (79.2% [38/48] vs. 12.5% [6/48], *p < *0.0001). In September 2015, lusutrombopag was approved by the Japanese Ministry of Health, Labor, and Welfare to treat thrombocytopenia in CLD patients receiving a treatment to improve based on L-PLUS 1 study [16]. In another placebo-controlled, double-blind, randomized clinical study in its phase 3, L-PLUS 2, the efficacy of lusutrombopag was evaluated in patients who were not Japanese. The results documented an evidently higher percentage of patients requiring neither pre-procedure transfusions of platelets nor rescue treatment for bleeding was significantly higher in the lusutrombopag group vs. the placebo group [64.8% (70/108) vs. 29.0% (31/107); *p < *0.0001]. Lusutrombopag was approved for marketing in the United States and the European Union based on L-PLUS 1 and 2 studies [17, 18]. The present study further demonstrated that in Chinese patients with thrombocytopenia and CLD, the proportion of responders (PLT ≥ 50 × 10^9^/L with an increase from baseline of ≥ 20 × 10^9^/L and not received rescue therapy) on Day 8 was significantly higher in the lusutrombopag group relative to the placebo group [43.2% (19/44) vs. 4.5% (1/22); *p* = 0.0011]. Similar to the clinical significance of the two phase 3 studies mentioned above, the primary endpoint of this study can also be used to evaluate the pre-procedure status of patients reflect the pre-procedure clinical benefits of patients to a certain extent. What is more, the clinical efficacy of lusutrombopag was proved consistently and clearly by the studies conducted in different countries.

Furthermore, the proportion of patients with PLT ≥ 50 × 10^9^/L on or after Day 8 and within 2 days before procedure was used as a surrogate indicator in this study, which indirectly verified the proportion of patients who should theoretically need platelet transfusion according to the platelet transfusion standard [6, 7]. The results suggested that lusutrombopag could significantly reduce the need for preoperative platelet transfusion by 54.5% in these thrombocytopenia patients with CLD before the invasive surgery. In fact, although platelet transfusion before surgery is allowed, only two patients (placebo group) in our study received platelet transfusion, due to the extreme donor shortage and concerns about transfusion risks. Therefore, considering the reality of the use of platelet products in China, it is not feasible to use platelet transfusion as the evaluation endpoint before surgery in this clinical trial.

In terms of safety, several randomized controlled studies and real-clinical evidence have suggested that lusutrombopag was well tolerated, being the same as that seen in this study [13, 14, 19, 20]. The occurrence of events related to bleeding in the lusutrombopag group was less relative to the placebo group (6.8% vs. 13.6%, respectively). Similar results were observed in the Japanese L-PLUS 1 study and the global L-PLUS 2 study. However, the incidence of bleeding-related events among patients treated with lusutrombopag was even lower (only 2.8%) in the L-PLUS 2 study, probably attributed to the rise in lower bleeding risk invasive procedures such as gastrointestinal endoscopy. And more patients received invasive procedures with a high risk of bleeding in the L-PLUS 1 study, like radiofrequency ablation; hence, the incidence of bleeding-related events among patients treated with lusutrombopag was up to 14.6%. Similar to the L-PLUS 1 study, procedures with a medium or high risk of bleeding, namely TAE/TAI/TACE, were the main type of invasive procedures performed on patients treated with lusutrombopag in our work. Hence the incidence of bleeding-related events here was slightly more than that in the L-PLUS 2 study. Moreover, it is not difficult to see that more subjects with high bleeding risk were enrolled in the Chinese study, which could better reflect the protective role of raising platelets through lusutrombopag treatment in the invasive surgery. It also suggests that invasive surgery with different bleeding risks will not affect the efficacy and safety of lusutrombopag.

Additionally, patients with liver cirrhosis are often associated with a potentially increased risk of vein thrombosis, and the use of TPO-RAs may further increase the risk of thrombosis [21]. Therefore, TPO-RA-related venous thrombosis has always been the key concerns for clinicians and may limit the use of TPO-RAs in clinical practice [22]. The ELEVATE study assessing the eltrombopag efficacy for increasing PLT and diminishing the necessity for transfusions of platelets in patients with thrombocytopenia and CLD receiving an invasive elective procedure was stopped early on account of an elevated thrombotic event frequency. Thrombotic events of the portal venous system occurred in 6 patients receiving eltrombopag, and five out of those with portal vein thrombosis experienced the event at PLT higher than 200 × 10^9^/L [23]. Another meta-analysis based on the randomized controlled trials of eltrombopag and avatrombopag (the only TPO-RAs receiving approval for treating CLD with thrombocytopenia in China) showed that the occurrence of portal vein thrombosis in patients having been administered eltrombopag or avatrombopag was notably higher relative to the placebo (OR = 3.36, 95% CI 1.07–10.59, *p* = 0.038), indicating that the above TPO-RAs are associated with portal vein thrombosis, which might bring potential negative effects for CLD patients in clinical application [24]. In the current study, no thrombosis of the portal vein was observed, and only one patient in the group treated with lusutrombopag experienced a brachiocephalic vein thrombosis which was assessed by ultrasonic examination to be mild. The most recent PLT before this thrombotic event was 42 × 10^9^/L, and the maximum PLT was 62 × 10^9^/L throughout the study, which indicated that the event was deemed irrelevant to the PLT elevation. Meanwhile, accounting for the patient's medical history of catheter placement in the left upper arm before the invasive procedure, the thrombotic events were not related to lusutrombopag.

It is worth noting that, unlike in Japan, Europe or America, hepatitis B is the main type of CLD in China, and the number of patients suffering from hepatitis B is about three times that of hepatitis C [25]. In this study, 86.4% of patients had hepatitis B at baseline, while the proportion of hepatitis B patients in the L-PLUS 1 and 2 studies did not exceed 20%. Therefore, this study demonstrated new insights into the efficacy and safety of lusutrombopag and provided more important guidance signification to the clinical practice in the treatment of HBV infected patients with cirrhosis, especially in China.

The administration regimen of lusutrombopag in this study was another noteworthy difference from L-PLUS 1 and 2 studies. To prevent the potential risk of thrombosis due to an excessive increase in platelets after the administration of lusutrombopag, the dose-stopping rule was implemented for avoiding platelet-overshooting in the L-PLUS 1 and L-PLUS 2 studies. As an inference result of the previous studies, it is possible that there is little difference in the probability of PLT higher than 200 × 10^9^/L without the dose-stopping rule in the course of seven-day treatment of lusutrombopag, and monitoring of platelet in patients administered lusutrombopag was not necessary [26]. This study further confirmed the conclusion that the risk of platelet counts exceeding 200 × 10^9^/L with a fixed seven-day dosing regimen is low, and additional platelet monitoring is not needed during the administration of lusutrombopag. The result could also give a new insight to physicians outside China. In addition, the pharmacokinetic profile of lusutrombopag was also analyzed in this study, and the result shows that the pharmacokinetics of lusutrombopag in the Chinese population are similar to those in other ethnic groups (data not shown).

A few limitations emerge in this work. First, while patients with Child–Pugh liver disease of class C that can be hospitalized at least between days 5 and 10 was one of the inclusion criteria, no such patients actually participated in the randomization. A study describing the pharmacokinetic characteristics of lusutrombopag showed a lower median AUC (area under the plasma concentration–time curve) of lusutrombopag in patients with Child–Pugh liver disease of class C was lower vs. the AUC in patients with Child–Pugh class A or B liver disease [27]. However, the sample size of patients with Child–Pugh class C liver disease in that study was relatively small, while *C*_max_ and AUC_0-τ_ overlapped between patients with Child–Pugh class A, B, or C liver disease. The data of patients with severe disease of the liver (Child–Pugh class C) were limited, and lusutrombopag should be used with caution. Second, substantial numbers of studies have confirmed the clinical efficacy of raising PLT and diminishing the necessity of pre-procedure platelet transfusion of lusutrombopag, but the crux lies in whether it can reduce the perioperative bleeding risk of patients [28]. The present study, along with the previous phase 3 studies, only described the patient percentage necessitating rescue treatment for bleeding and bleeding-related AEs, therefore warranting future probing to clarify the ability of lusutrombopag to diminish the risk of bleeding directly.

In conclusion, for the treatment of patients with CLD having thrombocytopenia, who plan to undergo invasive elective treatment, once-daily intake of lusutrombopag at 3 mg continuously for seven days can effectively raise PLT to meet the criteria of invasive procedures, thus avoiding pre-procedure platelet transfusion without additional safety problems. Crucially, lusutrombopag could be a safe, effective, and reliable method for such patients being treated with an elective invasive approach in China.

## Supplementary Information

Below is the link to the electronic supplementary material.

Supplementary file2 (DOCX 22 kb)

Supplementary file3 (DOCX 44 kb)

Supplementary file1 (TIF 8508 kb)
