# Supplementary material for: Lusutrombopag for thrombocytopenia in Chinese patients with chronic liver disease undergoing invasive procedures
Source: Hepatol Int. 2022 Oct 18;17(1):180–9. doi: 10.1007/s12072-022-10421-9 (PMC9895009; doi:10.1007/s12072-022-10421-9)
Supplement: Supplementary file 1 — Supplementary file2 (DOCX 22 kb) [file 12072_2022_10421_MOESM1_ESM.docx]

**Supplementary material 2** **Inclusion and exclusion criteria**

Inclusion criteria

| Patients who met all of the following criteria were eligible for inclusion in the study: |
| --- |
| 1. Able to understand the study and comply with all the study procedures，willing to provide written informed consent prior to screening; 2. Male or female, eighteen years of age or older at the time of signing informed consent; 3. Chronic liver disease  - patients of Child-Pugh class C, an be hospitalized at least between days 5 and 10  1. Platelet count <50 × 10^9^/L at baseline on day 1 prior to randomization 2. Undergoing an elective invasive procedure that:  - was likely to require platelet transfusion - was expected to be performed between days 9 and 14 - did not include laparotomy, thoracotomy, craniotomy, open-heart surgery, organ or partial organ resection (however, biopsy and other types of tissue removal were allowed if the risk of bleeding and invasiveness was considered comparable or lower than that of those procedures in the list of example procedures)  1. Eastern Cooperative Oncology Group (ECOG) performance status (PS) grade of 0 or 1; 2. In the opinion of the investigator, was able to meet the requirements of the study; 3. Male patients who were sterile or who agreed to use an appropriate method of contraception (including use of a condom with spermicide) from screening to completion of the post-treatment period; 4. Female patients who were not post-menopausal or surgically sterile had to agree to use a highly effective contraception (including contraceptive implant, injectable contraceptive, combination hormonal contraceptive[including vaginal ring], intrauterine contraceptive device, or vasectomized partner) from screening to completion of the post-treatment period. Barrier method with or without spermicide, double-barrier contraception and oral contraceptive pill were insufficient methods on their own. |

Exclusion criteria

| Patients with any of the following conditions were excluded from the study: |
| --- |
| 1. Any of the following diseases:  - hematopoietic tumor; - aplastic anemia; - myelodysplastic syndrome; - myelofibrosis; - congenital thrombocytopenia; - drug-induced thrombocytopenia; - immune thrombocytopenia; - generalized infection requiring treatment except for viral liver disease.  1. Any solid malignant tumor if:  - the patient required systemic chemotherapy, targeted therapy, immunotherapy, Chinese herbal therapy or radiotherapy for that malignant tumor during the study; - the malignant tumor was associated with nodal metastasis, distant metastasis, or invasion of the surrounding organs; - the exceptions were:   - a malignant tumor that was the treatment target of the primary invasive procedure;   - nonmelanoma skin cancer, intramucosal cancer, or carcinoma in situ not requiring treatment during the study.  1. History of liver transplantation; 2. Any of the following at screening:  - symptoms of hepatic encephalopathy with Child-Pugh hepatic encephalopathy score of 3 (occasionally coma), regardless of treatment - uncontrolled by drugs - total Bilirubin＞3mg/dL  1. Past or present thrombosis or prothrombotic condition (eg, cerebral infarction, myocardial infarction, angina pectoris, coronary artery stent placement, angioplasty, coronary artery bypass grafting, congestive heart failure [New York Heart Association Grade III/IV], arrhythmia known to increase the risk of thromboembolic events [eg, atrial fibrillation], pulmonary thromboembolism, deep vein thrombosis, or disseminated intravascular coagulation syndrome); 2. History or presence of disease associated with a risk of bleeding (eg, coagulation factor deficiency or von Willebrand factor deficiency); 3. History or presence of any of the following diseases:  - congenital thrombotic disease (eg, antithrombin deficiency, protein C deficiency, protein S deficiency, or coagulation factor [factor V Leiden] mutation); - acquired thrombotic disease (eg, antiphospholipid antibody syndrome, paroxysmal nocturnal hemoglobinuria, hyperhomocysteinemia, or increased factor VIII); - Budd-Chiari syndrome.  1. Portal vein tumor embolism; 2. Portal vein thrombosis based on ultrasound, computed tomography (CT), or magnetic resonance imaging (MRI) within 28 days prior to randomization or a history of portal vein thrombosis; 3. Absence of hepatopetal blood flow in the main trunk of the portal vein as demonstrated by Doppler ultrasonography within 28 days prior to randomization; 4. Untreated gastro-esophageal varices that were bleeding or required treatment based on upper gastrointestinal endoscopy within 180 days prior to randomization (except for patients in whom the primary invasive procedure was for the treatment of gastro-esophageal varices); 5. Bleeding score at randomization ≥ Grade 2 according to the World Health Organization (WHO) Bleeding Scale; 6. Any of the following drugs or therapies within 90 days prior to randomization:  - anticancer drugs except for transcatheter arterial chemoembolization (TACE) and lipiodolization; - interferon preparations; - radiation therapy; - exsanguination; - other thrombopoietin (TPO) receptor agonist; - IL-11; - any investigational agent.  1. Any of the following invasive procedures within 90 days prior to randomization:  - laparotomy, thoracotomy, craniotomy, open-heart surgery; or procedures involving any organ resection or any partial organ resection (tissue resection associated with an endoscopic examination was permitted); - partial splenic embolization.  1. Any invasive procedure (except for the treatment of gastro-esophageal varices) within 14 days prior to randomization; 2. Blood transfusion (except for red blood cell products and albumin preparations) within 14 days prior to randomization; 3. Patients who had received lusutrombopag before; 4. Known allergy to the test drug or any of its excipients 5. Known to be positive for the human immunodeficiency virus antigen/antibody at screening; 6. Patients with known or suspected ongoing, active alcohol or substance abuse; patients with a recent history who the investigator felt were able to comply with the study procedures and medications were allowed to participate; 7. Pregnant or lactating females; 8. Considered ineligible by the investigator for any other reason. |
