# Supplementary material for: Lusutrombopag for thrombocytopenia in Chinese patients with chronic liver disease undergoing invasive procedures
Source: Hepatol Int. 2022 Oct 18;17(1):180–9. doi: 10.1007/s12072-022-10421-9 (PMC9895009; doi:10.1007/s12072-022-10421-9)
Supplement: Supplementary file 2 — Supplementary file3 (DOCX 44 kb) [file 12072_2022_10421_MOESM2_ESM.docx]

**Supplementary material 3 Trial profile**


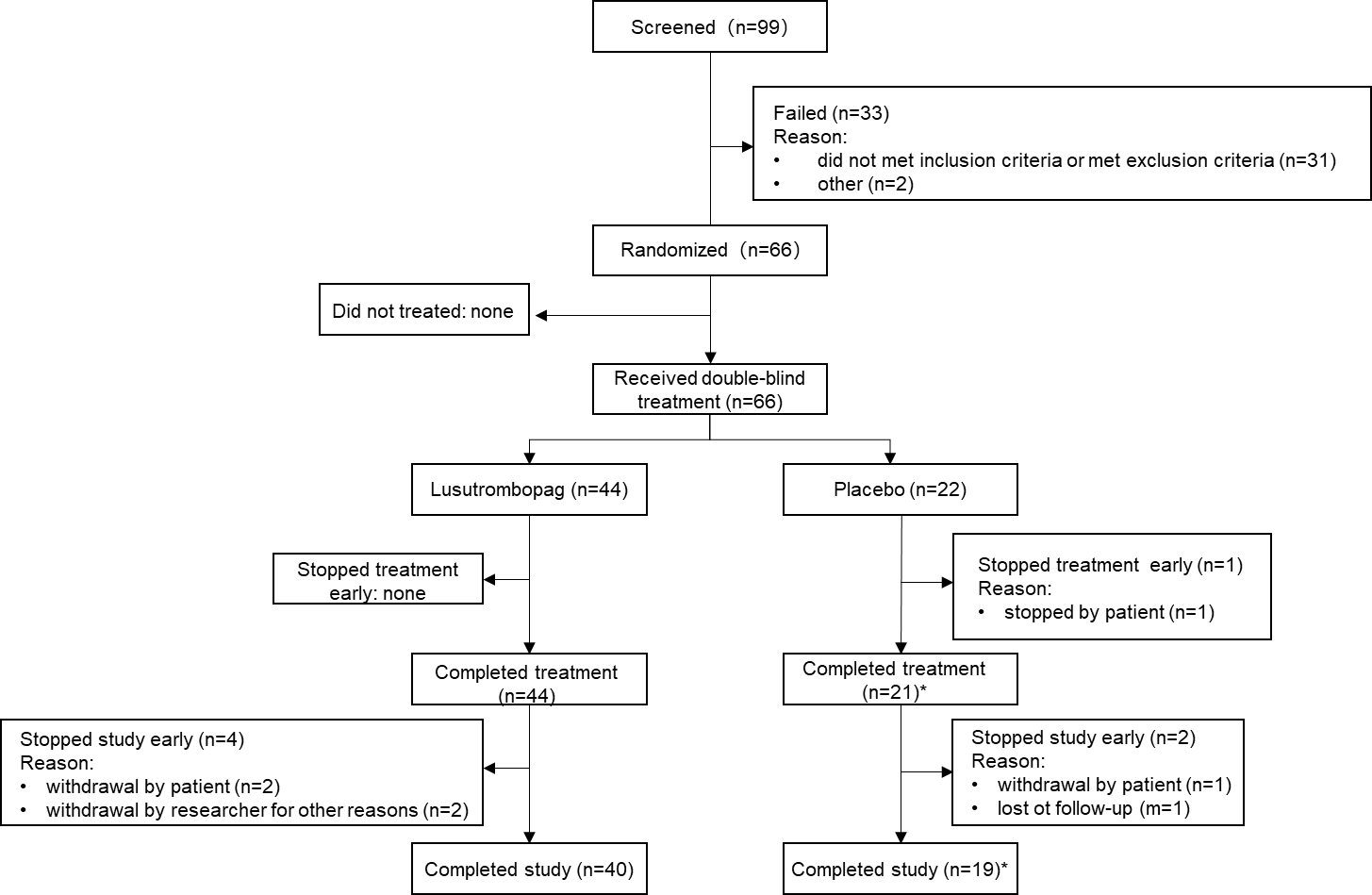


*Including the patient who took only 6 times due to drug loss
