# Supplementary figures and images for: Lusutrombopag for thrombocytopenia in Chinese patients with chronic liver disease undergoing invasive procedures
Source: Hepatol Int. 2022 Oct 18;17(1):180–9. doi: 10.1007/s12072-022-10421-9 (PMC9895009; doi:10.1007/s12072-022-10421-9)

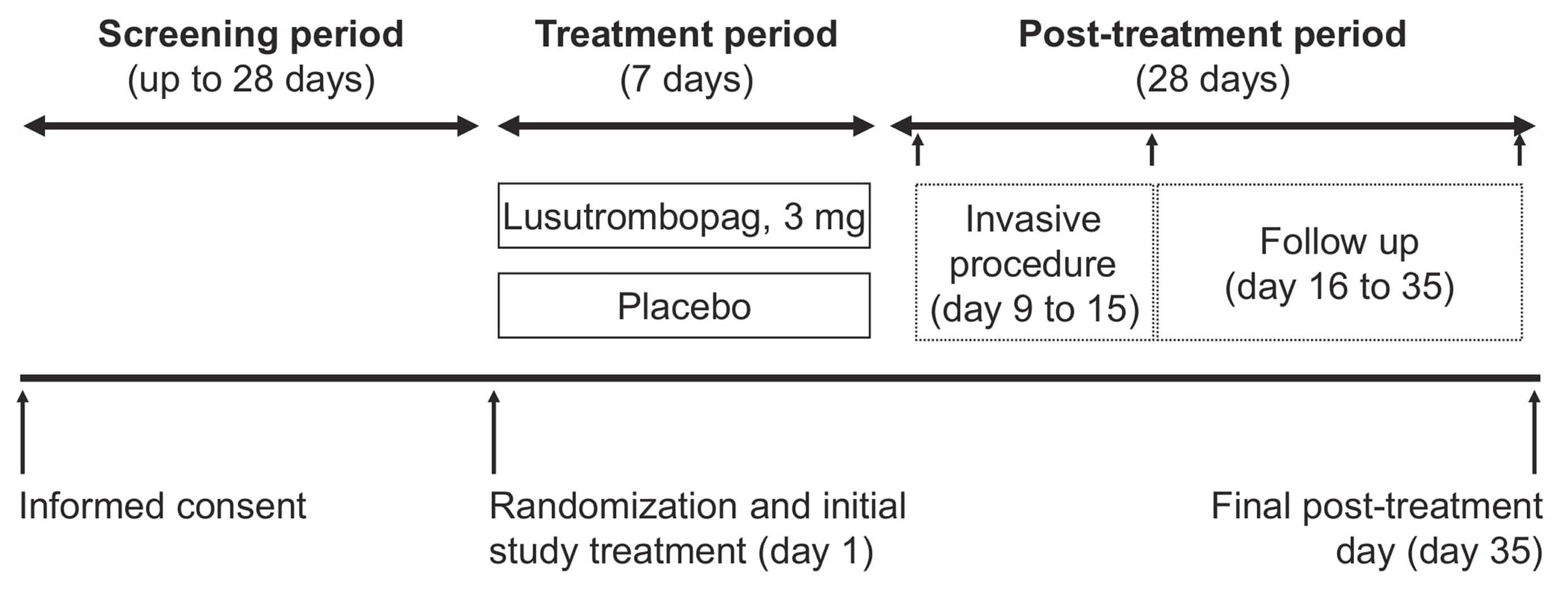

Supplement: Supplementary file 3 — Supplementary file1 (TIF 8508 kb) [file 12072_2022_10421_MOESM3_ESM.tif]
